# What can we learn from experience? Impact of healthcare provider effects in the total or partial knee arthroplasty trial (TOPKAT)

**DOI:** 10.1186/1745-6215-16-S2-P3

**Published:** 2015-11-16

**Authors:** Jonathan Cook, Graeme MacLennan, David Murray, Andrew Price, Ray Fitzpatrick, Andrew Carr, Marion Campbell, Helen Campbell, Nigel Arden, Cushla Cooper, Loretta Davies, David Beard

**Affiliations:** 1 University of Oxford, Oxford, UK; 2 University of Aberdeen, Aberdeen, UK

## 

Conducting multi-centre randomised controlled trials (RCTs) of surgical procedures raises specific issues in design, analysis and reporting. Interventions evaluated are usually complex and can be affected by factors such as surgical skill, decision making, preoperative and postoperative care. Variation between participating surgeons may have an important impact on the treatment effect.

The effect of healthcare provider (surgeon experience) was examined in the year 1 analysis of the TOPKAT study. This multi-centre, randomised controlled trial aims to assess the clinical and cost effectiveness of total or partial knee replacements for medial compartment osteoarthritis. The study has a combined expertise (7 sites)/equipoise (20 sites) design involving 64 surgeons in 27 secondary care orthopaedic units from across the UK. Both interventions evaluated are established and well-documented procedures. A minimal standard of experience was specified for entry into the study. Difference in prior surgeon experience with number of TKR procedures performed, median (IQR) 342 (296,1000) compared to UKR 150 (90,225), reflected that it is a more established procedure.

The influence of healthcare provider on treatment effect in the TOPKAT study will be reported. The importance of considering the impact of participating centres in the design, analysis and reporting of surgical RCTs will be discussed.

